# Coronavirus Disease (COVID-19): Challenges and Opportunities

**DOI:** 10.1017/dmp.2020.341

**Published:** 2020-09-10

**Authors:** Gholamreza Farnoosh, Gholamhossein Alishiri, Alireza Jalali Farahani, Nasirudin Javidi, Zahra Farhangi, Mohammadkarim Bahadori, Ramin Ravangard

**Affiliations:** Applied Biotechnology Research Center, Baqiyatallah University of Medical Sciences, Tehran, Iran; Chemical Injuries Research Center, Systems Biology and Poisonings Institute, Baqiyatallah University of Medical Sciences, Tehran, Iran; Trauma Research Center, Baqiyatallah University of Medical Sciences, Tehran, Iran; Behavioral Sciences Research Center, Life Style Institute, Baqiyatallah University of Medical Sciences, Tehran, Iran; Health Research Center, Baqiyatallah University of Medical Sciences, Tehran, Iran; Health Management Research Center, Baqiyatallah University of Medical Sciences, Tehran, Iran; Health Human Resources Research Center, School of Management and Information Sciences, Shiraz University of Medical Sciences, Shiraz, Iran

Since December 2019, the world health care community has faced the 2019 coronavirus disease (COVID-19) outbreak caused by severe acute respiratory syndrome coronavirus 2 (SARS-CoV-2).^1^ Coronavirus is a large family of viruses that can cause respiratory infections ranging from the common cold to more severe illnesses such as the Middle East respiratory syndrome 2 (MERS) and Severe Acute Respiratory Syndrome (SARS). The outbreak of this new virus began in December 2019 in Wuhan, China.^2^ The virus seems to be extremely contagious and has rapidly spread throughout the world.^3^ The virus is a submucosal infectious agent that only multiplies in the living cells of an organism. There are different types of viruses. They can infect animals, plants, and microorganisms, including bacteria and the ancients.^4^

MERS is also a viral respiratory disease that was first reported in Saudi Arabia in 2012 and has spread to several other countries. Moreover, SARS is a viral respiratory disease caused by a coronavirus called the *coronavirus associated with SARS* (SARS-CoV).^5^

The virus that causes COVID-19 is easily spread in areas where there is a living cell. This local spread means that people may be directly infected with the virus or they can be carriers of it.^5^

As far as research is concerned, leading scientists are looking for the treatment of COVID-19. In Iran, the Baqiyatallah University of Medical Sciences has started a large scientific research on the rapid diagnosis, vaccine, and drug discovery with the help of a team of physicians and researchers and has achieved promising results, so far.

## CHALLENGES

**FIGURE 1:**
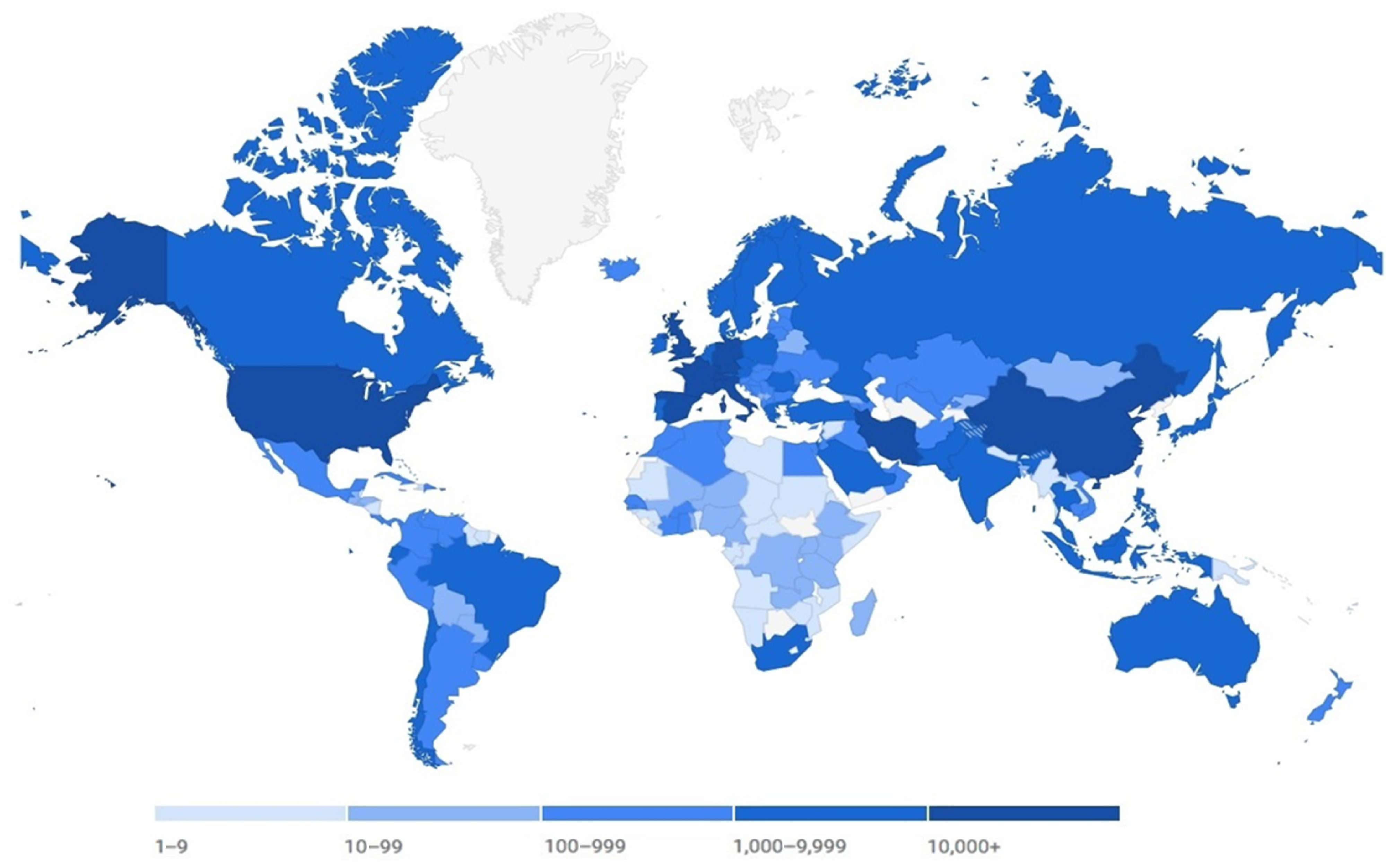
The Prevalence of COVID-19 in Countries Around the World by March 25, 2020 *(newatlas.com)*.

The virus transcended China’s borders very quickly and spread to 212 countries by May 5, 2020. This process is called *human-to-human transmission*, which contributes to the virus spread and expansion.^6^ The countries with the highest prevalence of COVID-19 confirmed by the World Health Organization (WHO) include China, United States, Italy, Spain, Germany, France, Iran, and the United Kingdom. With the rapid global spread of COVID-19, which has affected 212 countries worldwide by May 5, 2020 (3 659 103 people affected, 252 573 deaths, and 1 203 404 persons recovered), the world is getting closer to the pandemic of COVID-19 more than ever (Figure 1).

The symptoms of the virus vary from mild to severe. Its symptoms include fever, cough, and difficulty with breathing.^7-10^ Anxiety is also considered a psychological symptom and a common symptom in patients with chronic respiratory disorders and can significantly reduce patients’ quality of life.^11-13^

Little research has been conducted on the experience of anxiety in patients with COVID-19.^14^ In fact, anxiety in such patients is mostly due to the unknown disease and the cognitive ambiguity of people about the virus that this anxiety can also disrupt the lives of families and couples.^15-17^ Fear of the unknown reduces the perception of immunity in humans and has always been a source of anxiety for mankind. The lack of scientific information about COVID-19 also exacerbates this anxiety.^18,19^ Therefore, stress and anxiety can weaken the immune system.^20,21^ As a result, people should learn strategies and skills to cope with anxiety, which can enhance human empowerment. Given the rapid spread of the disease, research with regard to the prevention and treatment is needed to improve people’s quality of life and community health.^22-24^ The feeling of insecurity caused by anxiety is treated with psychotherapeutic approaches such as emotionally focused therapy.^25,26^

## OPPORTUNITIES

This virus, along with the damages it has caused, also provided opportunities for people to develop their existential and skill-building capacities and has led to achievements such as upgrading hardware and software capacities, improving the knowledge of specialists, adhering to health and hygiene principles by the general public, enhancing the spirit of empathy between people and authorities, understanding the importance of the medical staff’s efforts, publishing scientific articles and reaching the boundaries of knowledge, identifying strengths and weaknesses in various areas (especially in the field of interventions in crises), increasing public awareness in the medical field (especially about communicable diseases), and enhancing the people and authorities’ sense of responsibility and lack of excessive trust in the cyberspace.^27^

Its psychological achievements also include improving emotional regulation in individuals, identifying individuals’ existential capacities in times of crisis, controlling anxious beliefs, improving psychological adjustment, recalling death anxiety, remembering God and seeking refuge in God, enhancing creativity and innovation, individual self-assessment in the field of courage/fear and indifference/responsibility, measuring human patience and resilience, better understanding of religious beliefs, better understanding of the value of health, distinguishing opportunism and profit- and fame-seeking from truth-seeking, human attention to family and friends, empowering empathy, and creating home opportunities and jobs due to home quarantine.^28-30^

In the end, it should be stated that, although it is true according to the Holy Quran that “Whatever benefit comes to you, it is from Allah” (Verse 79, Surah AN-NISA),^31^ God also says elsewhere in the Quran that “ It may be that you dislike a thing while it is good for you, and it may be that you love a thing while it is evil for you, and Allah knows, while you do not know” (Verse 216, Surah Al-Baqara). Also, God says “Do not despair of the mercy of Allah; surely Allah forgives the faults altogether; surely, He is the Forgiving of the Merciful” (Verse 53, Surah Az-Zamar).^31^

According to the abovementioned quotes and also regarding the fact that, in the Holy Quran, it is believed that after each and every difficulty comes relief (Verse 94, Surah Al-Sharh), it can be concluded that these difficult days will definitely pass by, and ease and relief will come back to human beings’ lives.

Therefore, it seems that, under such circumstances, the human duty is to deal with the damage caused by COVID-19, as well as to enhance the individuals’ scientific and psychological capabilities. So, it is best to focus on opportunities to grow and improve the existential capacities in times of crisis. Overall, given that Iran, with a population of 81 million and having a strategic position in the world, faces the most severe economic sanctions imposed, it has been able to cope with the disease and has achieved some success through the empathy between the people and the authorities, resulting in 79 379 recoveries and improvements as of May 5, 2020.
